# Genetic diversity and population structure of 93 rice cultivars (lines) (*Oryza sativa* Xian group) in Qinba in China by 3 types of genetic markers

**DOI:** 10.1186/s12864-022-08707-1

**Published:** 2022-08-02

**Authors:** Yu Zhang, Qiaoqiao He, Xixi Zhou, Shimao Zheng, Yewen Wang, Peijiang Li, Yuexing Wang

**Affiliations:** 1 Shaanxi University of Technology, Hanzhong, 72300 Shaanxi China; 2 Shaanxi Province Key Laboratory of Bio-resources, Hanzhong, 72300 Shaanxi China; 3 QinLing-Bashan Mountains Bioresources Comprehensive Development C. I. C., Hanzhong, 72300 Shaanxi China; 4 Qinba State Key Laboratory of biological resources and ecological environment, Hanzhong, 72300 Shaanxi China; 5 Shaanxi Rice Research Institute, Hanzhong, 723000 Shaanxi China; 6 College of Agronomy, Xinjiang Agricultural University, Urumqi, 830052 China

**Keywords:** Indica rice, Phenotypic traits, SSRs, SNPs, Genetic diversity, Population structure

## Abstract

**Background:**

The Qinba region is the transition region between Indica and Japonica varieties in China. It has a long history of Indica rice planting of more than 7000 years and is also a planting area for fine-quality Indica rice. The aims of this study are to explore different genetic markers applied to the analysis population structure, genetic diversity, selection and optimization of molecular markers of Indica rice, thus providing more information for the protection and utilization on germplasm resources of Indica rice.

**Methods:**

Fifteen phenotypic traits, a core set of 48 SSR markers which originated protocol for identification of rice varieties-SSR marker method in agricultural industry standard of the People's Republic of China (Ministry of Agriculture of the PRC, NY/T1433-2014, Protocol for identification of rice varieties-SSR marker method, 2014), and SNPs data obtained by genotyping-by-sequencing (GBS, *Nla*III and *Mse*I digestion, referred to as SNPs-*Nla*III and SNPs-*Mse*I, respectively) for this panel of 93 samples using the Illumina HiSeq2000 sequencing platform, were employed to explore the genetic diversity and population structure of 93 samples.

**Results:**

The average of coefficient of variation (*CV*) and diversity index (H_e_) were 29.72% and 1.83 ranging from 3.07% to 137.43%, and from 1.45 to 2.03, respectively. The correlation coefficient between 15 phenotypic traits ranged from 0.984 to -0.604. The first four PCs accounted for 70.693% phenotypic variation based on phenotypic analysis. A total of 379 alleles were obtained using SSR markers, encompassing an average of 8.0 alleles per primer. Polymorphic bands (PPB) and polymorphism information content (PIC) was 88.65% and 0.77, respectively. The Mantel test showed that the correlation between the genetic distance matrix based on SNPs-*Nla*III and SNPs-*Mse*I was the largest (R^2^=0.88), and that based on 15 phenotypic traits and SSR was the smallest (R^2^=0.09). The 93 samples could be clustered into two subgroups by 3 types of genetic markers. Molecular variance analysis revealed that the genetic variation was 2% among populations and 98% within populations (the Nm was 0.16), Tajima’s D value was 1.66, the FST between the two populations was 0.61 based on 72,824 SNPs.

**Conclusions:**

The population genetic variation explained by SNPs was larger than that explained by SSRs. The gene flow of 93 samples used in this study was larger than that of naturally self-pollinated crops, which may be caused by long-term breeding selection of Indica rice in the Qinba region. The genetic structure of the 93 samples was simple and lacked rare alleles.

## Key message

It was found that there was a significant correlation between the genetic distance obtained by the two types of SNPs markers, while the lowest correlation occured between the genetic distance of phenotypic traits and SSR data. The population genetic variation explained by SNPs was larger than that explained by SSRs among DNA molecular markers. Bayesian clustering algorithm was superior to the other two clustering methods. The genetic structure of 93 samples representative of the diversity present in Qinba area in China of *Oryza sativa* Xian group was simple and lacked rare alleles.

## Background

According to the origin and the history of rice cultivate in China, the two major types of *Oryza sativa* L. are classified as *O. sativa* L. subsp. *hsien* Ting and *O. sativa* L. subsp. *keng* Ting proposed by Ding Y [1, 2], and the naming method of *O. sativa* L. subsp. *indica* Kato (also known as *O. sativa* Xian group) and *O. sativa* L. subsp. *japonica* Kato (also known as *O. sativa* Xian group ) for *O. sativa* L. proposed by Kato was used internationally at the present [3], in which Indica rice is distributed mainly in the southern Qinling Mountains in China. The Qinba area is the climate transition area between the northern and southern areas as well as the transition area from Indica rice to Japonica rice, which is also the most suitable planting area for Indica rice in China. Germplasm resources form the basis of all breeding work; the analysis of genetic diversity and genetic structure is beneficial to mining excellent breeding materials and improving breeding efficiency. Particularly, in-depth genetic dissection of Indica rice germplasm resources have not been conducted. The population genetic structure is the non-random distribution of genes or genotypes in space and time, including genetic variations within populations and genetic differentiation between populations. Population structure analysis is essential to explore the biological adaptability, population formation process, evolutionary mechanism, protection, and development of biological resources. At the same time, populations with identical or similar genetic backgrounds is most suitable for genome-wide association studies (GWAS), therefore, the study of population genetic structure plays an important role in the field of biology, in which the selection of genetic markers is the top strategic priority, ranging from earlier morphological markers to more recent different types of DNA molecular markers [4–7]. The Indica rice genome has simple sequence repeats (SSRs) that span approximately 10-50 kb [8, 9]. In the last few decades, SSR molecular markers have become important tools in the field of biology, particularly in terms of population structure, genetic mapping, and other related fields, SSR markers have also become the designated markers of the International Fingerprint Mapping Center [10–12]. These are employed in judicial identification, identification of new varieties of plants, such as rice, rape, and corn [13–18]. SSR markers are also used in DNA fingerprinting for breed protection [19]. However, SSR markers are scarce, show unbalanced distribution in the genome, have weak electrophoretic resolution, and are relatively time-consuming and labor-intensive to study, and thus it is difficult to construct high-density genetic maps. With the recent development of next-generation sequencing technology, most biological studies have rapidly improved, in particular, the use of single nucleotide polymorphisms (SNPs) based on genome-wide scans. With the release of extensive rice genome sequencing data, one SNP in every hundred base pairs or even every dozens of base pairs has been identified, indicating that there are numerous SNPs in the rice genome [6, 20]. A small number of SNPs can be used to resolve many problems, so the sequencing technology was born based on simplified genome by restriction site-associated DNA (RAD) tags [21]. The frontrunner among these technologies is genotyping-by-sequencing (GBS), which has recently gained attention because it utilizes methylation-sensitive restriction endonucleases (type II enzyme), thereby avoiding repetitive regions of the genome (methylated regions). GBS technology can rapidly identify high-density polymorphisms, especially SNPs [22]. In this study, two type II enzymes (*Nla*III and *Mse*I) were selected by simulated whole-genome enzyme digestion, which generated RAD tags for sequencing to obtain SNPs datasets, referred to as SNPs_*-Nla*III_ and SNPs_*-Mse*I_*,* respectively. Simultaneously, a core set of SSR markers from NY/T1433-2014 [23] that originated in the Agricultural Standards of the People's Republic of China and 15 phenotypic traits were employed to explore gene flow and population genetic structure of 93 samples and to provide reference for future research studies using different genetic markers employed in related fields.

## Results

### Phenotypic traits diversity and cluster analysis

#### Phenotypic diversity analysis

Data of the 15 phenotypic traits of the 93 samples are summarized in Table 1. The basic statistical analysis and diversity of the 15 phenotypic traits based on phenotypic data is shown in Table 2. The coefficient of variation (*CV*) was 29.72% on average and ranged from 3.07% (brown rice rate) to 137.43% (chalkiness). The average diversity index (*H*_*e*_) was 1.92 and ranged from 1.55 to 2.08, with brown rice rate and chalkiness having lower *H*_*e*_, indicating that there were relatively few phenotypes in these two traits. Overall, the Indica rice materials tested had relatively more phenotypes on these 15 traits; the distribution in each phenotype was uneven Tables 3 and 4.

**Table 1:** Phenotypic data of 93 samples

Name	Type	1	2	3	4	5	6	7	8	9	10	11	12	13	14	15
W1	R	115	128.2	46.26	2.58	6.17	28.95	241.36	217.64	28.57	79.14	67.47	58.60	17.50	4.00	2.40
W298	S	115	128.2	46.26	2.58	6.17	28.95	241.36	217.64	28.57	79.14	67.47	58.60	17.50	4.00	2.40
W2	R	114	121.8	53.3	2.46	7.83	27.94	219.00	181.75	28.82	92.16	60.35	54.90	51.50	12.00	2.30
W300	S	81	111	47.38	1.96	5.50	25.42	184.07	177.53	24.78	77.77	58.56	57.00	100.00	79.80	2.40
W352	S	102	112.4	30.76	2.22	7.50	24.22	140.43	135.00	26.31	76.33	55.36	43.90	58.60	22.20	2.50
W353	S	115	127.8	42.62	1.96	6.82	28.30	128.45	112.14	31.81	77.24	64.10	46.30	54.40	15.1	2.3
W354	S	122	120.2	29.94	2.01	6.35	27.21	169.50	157.10	29.52	76.80	59.29	29.60	21.70	5.9	2.5
W355	S	117	117.6	45.82	2.11	8.13	23.93	162.30	146.11	27.34	75.68	63.34	51.00	3.30	0.9	2.9
W357	S	100	106	48.78	2.4	5.17	26.72	200.93	180.00	22.23	77.18	52.41	43.80	72.70	24.70	2.00
W359	S	96	135.8	32.28	2.48	6.67	25.98	213.56	188.56	24.14	77.90	59.42	58.70	23.10	7.30	2.40
W361	S	109	113.2	44.52	2.06	4.83	27.93	187.31	175.19	33.51	74.70	50.25	48.40	91.20	58.80	2.10
W366	S	105	124.6	40.42	1.72	9.00	23.71	159.72	133.33	19.46	69.03	50.18	49.10	97.20	79.00	1.50
W367	S	103	110	55.36	1.8	3.33	25.96	247.25	237.00	27.73	81.64	51.28	48.60	95.60	42.60	1.70
W369	S	105	109.2	37.22	2.1	6.33	24.41	221.87	195.00	20.51	77.39	49.92	37.30	18.40	5.40	2.40
W370	S	110	129.2	45.26	2.16	10.33	28.80	221.94	208.17	23.60	77.30	53.29	48.20	18.80	7.90	2.50
W375	S	85	98.6	36.3	2.02	6.67	25.90	231.06	214.29	19.30	77.54	50.08	41.40	38.90	13.20	2.30
W377	S	103	123	35.88	2.38	3.83	26.47	236.86	216.71	23.07	77.72	47.80	53.30	12.50	3.80	2.20
W380	S	100	98	36.76	2	7.17	25.96	216.39	196.94	20.21	77.23	48.00	24.60	49.80	18.60	2.20
W381	S	98	99.6	28.8	2	4.50	31.31	228.86	203.93	22.10	75.31	53.19	48.00	19.90	4.80	2.50
W3	R	116	133.2	45.34	2.38	6.33	29.28	216.14	201.36	36.22	79.87	51.44	28.90	37.60	9.30	2.40
W4	R	115	121.8	46.98	2.14	7.17	27.94	176.63	168.94	30.67	79.44	60.83	60.30	23.00	4.90	2.40
W5	R	113	125.6	46	2.28	6.00	31.35	254.55	247.36	26.83	78.58	56.36	46.70	17.70	4.10	2.50
W666	R	105	109.8	39.5	1.68	10.17	22.25	127.20	122.50	23.10	79.66	56.12	55.60	2.80	0.70	2.40
W667	R	101	105	35.6	2.43	6.83	22.04	175.00	168.50	24.41	78.62	60.95	55.90	25.70	7.70	2.10
W668	R	109	116.4	39.14	2.2	8.17	25.90	142.90	130.59	30.20	77.87	63.30	62.60	10.50	2.50	2.40
W669	R	109	110.8	37.56	1.78	10.33	24.84	140.95	115.29	21.67	78.60	64.80	64.10	4.00	0.80	2.70
W670	R	104	132	38.52	2.26	5.50	26.93	176.40	166.28	32.35	77.20	53.32	50.00	56.80	15.6	2.4
W671	R	110	128.6	45.76	2.62	6.17	28.33	190.31	176.38	31.60	80.06	60.73	59.40	53.70	15.60	2.30
W672	R	104	121.8	41.98	2.12	6.83	23.69	143.73	127.64	34.36	78.55	60.60	59.90	7.10	1.10	2.60
W673	R	106	119.6	37.83	2.15	8.00	26.10	128.07	120.21	30.95	72.24	55.13	52.70	7.80	2.10	2.40
W674	R	110	135.8	43.4	2.08	5.17	28.33	190.31	176.38	34.66	76.61	58.74	58.30	13.80	3.10	2.50
W675	R	110	118.6	30.84	1.98	8.00	27.53	154.08	141.33	29.10	78.89	57.35	55.20	22.80	5.00	2.40
W676	R	109	106	30.12	2.2	6.17	25.65	165.25	153.00	23.64	76.92	54.06	53.90	1.20	0.30	2.10
W677	R	110	112.2	30.12	2.1	8.17	24.23	174.44	159.63	29.13	79.80	58.10	56.60	18.10	3.50	2.30
W678	R	110	112	39.24	2.14	10.83	24.91	131.57	111.38	30.99	80.68	58.13	53.10	10.60	1.90	2.60
W679	R	112	108.8	38.18	2.3	7.83	24.72	133.44	120.94	29.62	78.82	60.50	59.60	19.90	4.50	2.60
W680	R	107	121.6	43.76	2.08	6.83	27.08	151.26	141.86	31.99	78.55	63.99	61.40	28.30	7.60	2.70
W681	R	111	116.8	37.56	2.3	5.17	24.72	130.73	119.00	30.08	76.89	54.86	54.00	1.60	0.20	2.30
W684	R	111	116.6	36.6	2.3	7.33	26.56	168.31	163.13	31.26	79.58	61.82	60.00	5.80	1.70	2.30
W685	R	110	121.4	31.94	2.44	8.17	23.98	176.87	169.53	33.26	79.48	56.74	54.50	35.20	10.00	2.10
W686	R	110	121	38.8	2.48	6.83	24.97	184.50	172.92	25.40	78.90	60.28	59.80	23.40	5.00	2.00
W687	R	110	108.4	38.22	2.12	7.33	25.07	142.33	128.87	28.74	78.56	56.34	54.10	29.50	6.90	2.20
W688	R	108	114.6	43.2	2	7.83	26.27	147.40	139.27	34.58	78.12	60.78	59.80	13.10	2.30	2.60
W689	R	109	105.2	36.54	2.54	8.67	25.75	131.06	86.86	30.03	80.33	54.00	39.80	29.40	5.80	2.20
W690	R	113	104.8	33.14	2.04	10.17	24.06	96.70	84.85	26.11	76.74	59.39	58.10	4.40	0.80	2.90
W691	R	108	115.6	44.72	2.02	8.83	28.18	135.40	130.30	34.40	78.35	56.20	51.50	9.40	1.90	2.60
W692	R	108	116.8	45.8	2.66	6.50	26.02	262.85	212.18	24.50	78.72	65.00	62.70	3.70	1.10	3.00
W693	R	114	122	39.16	2.52	7.17	24.98	234.75	208.13	27.27	77.99	58.61	57.20	23.90	8.90	2.20
W694	R	110	103.8	33.52	1.6	10.67	23.33	137.54	129.04	22.59	80.13	56.70	48.00	9.60	3.00	2.60
W697	R	105	90.8	27.32	1.82	7.33	22.96	114.19	101.31	21.27	79.24	62.57	61.80	2.60	0.70	2.40
W698	R	107	115.8	41.6	2.04	9.67	25.65	158.88	153.17	25.75	79.04	59.82	58.70	14.20	3.70	2.40
W699	R	102	108.8	80.7	2.22	3.83	26.27	428.50	394.60	20.63	78.94	61.29	58.80	9.50	2.40	2.60
W6	R	117	125.2	44.12	2.32	6.17	23.90	236.15	211.31	29.18	82.15	56.04	47.20	25.60	5.20	2.20
W700	R	101	121.8	35.6	2.72	7.17	24.09	166.38	153.81	25.17	79.40	63.40	62.00	1.60	0.30	2.60
W701	R	113	141.2	45.16	1.68	9.50	28.70	188.70	181.15	22.20	77.79	60.04	58.60	17.50	4.00	2.40
W702	R	107	126.6	33.08	1.78	10.83	21.27	127.13	118.13	23.38	78.85	63.27	61.40	0.70	0.20	2.60
W703	R	107	108.6	35.32	1.66	8.00	23.84	162.30	147.04	20.43	79.58	63.86	63.50	2.50	0.40	2.70
W704	R	114	133.4	45.7	1.9	5.40	24.22	206.46	196.77	25.36	78.24	56.96	56.20	5.80	1.10	2.40
W707	R	106	116.6	38.02	1.76	8.33	23.91	141.78	134.67	18.61	77.21	62.43	61.80	5.00	1.10	2.90
W708	R	104	117.4	33.16	2.26	8.17	26.06	235.25	216.00	23.70	76.51	61.23	59.10	7.41	1.23	2.60
W710	R	104	116.4	30.1	1.86	7.33	23.69	177.88	168.25	29.56	81.48	60.29	59.30	91.10	23.20	1.80
W711	R	102	115	32.88	1.82	8.17	22.98	159.88	153.24	28.85	81.24	67.08	65.80	89.10	21.20	1.80
W713	R	108	114.2	35.7	1.64	7.67	24.48	232.19	201.19	20.37	79.93	64.68	64.20	1.20	0.20	2.80
W714	R	100	120.2	26.94	1.9	7.33	21.90	200.82	181.59	19.69	79.96	59.60	57.70	3.00	0.70	2.40
W715	R	104	118.6	35.08	1.8	8.50	26.41	233.60	219.87	22.28	78.34	62.90	62.70	2.00	0.50	2.70
W716	R	92	104.4	39.22	2.08	6.67	24.20	175.50	145.70	28.09	78.25	62.24	56.80	13.70	3.40	2.70
W717	R	105	135	41.06	2.32	6.67	28.69	192.38	180.15	25.76	79.01	63.85	57.50	52.50	14.50	2.30
W718	R	113	141.4	36.28	2.1	10.17	26.83	153.96	137.42	27.43	79.26	61.09	59.90	2.20	0.30	2.70
W719	R	97	125.4	32.66	1.88	7.83	22.45	145.20	138.60	28.62	79.48	60.49	53.40	0.70	0.10	3.10
W720	R	99	122.4	32.56	2.04	9.17	24.35	112.14	104.83	29.21	77.50	58.73	54.70	2.00	0.30	2.90
W721	R	105	98.2	35.22	2.4	5.67	25.36	183.46	178.54	27.12	77.43	56.51	52.60	48.70	39.20	2.20
W722	R	96	123.6	37.06	2.18	6.17	26.69	159.75	110.56	24.65	79.20	55.98	15.30	39.50	10.30	2.90
W723	R	105	129.2	37.64	2.62	6.67	28.06	255.29	245.93	22.96	80.44	62.13	60.80	14.00	4.20	2.30
W724	M	84	93.4	41.5	1.54	8.67	22.65	148.47	126.25	20.37	80.37	61.14	58.80	15.30	4.90	2.40
W725	M	89	99.2	44.26	1.74	7.67	23.71	196.28	182.00	24.47	83.39	62.30	51.30	13.40	3.70	2.40
W726	M	78	76.2	33.46	1.38	13.67	21.25	99.33	80.42	28.09	82.26	64.04	59.70	85.90	33.00	2.70
W727	M	80	88.6	40.52	1.76	8.00	23.10	120.29	112.19	29.70	79.21	60.38	56.20	30.68	12.79	2.50
W728	M	75	82.6	36.14	1.42	10.33	20.88	98.26	90.37	26.50	80.29	61.90	57.32	16.97	6.70	2.60
W730	M	86	95.6	36.22	1.42	11.33	22.60	128.52	124.67	24.01	77.68	53.86	40.40	59.50	22.50	2.30
W732	M	89	90.2	29.7	1.4	7.67	20.20	125.82	119.68	25.93	79.99	56.99	43.30	81.00	23.50	1.90
W733	M	81	75.8	29.3	1.28	10.83	18.17	70.53	63.07	28.11	80.07	58.88	52.40	80.80	26.90	2.50
W734	M	91	90.2	34.44	2.22	9.33	21.92	165.61	150.83	24.90	80.61	60.13	57.80	93.80	41.10	1.70
W735	M	87	89.2	37.42	1.54	9.67	20.91	131.59	128.12	26.67	79.86	58.57	43.30	89.90	30.70	2.00
W736	M	84	86	31.12	1.5	12.00	20.20	100.84	94.72	29.44	78.64	51.84	38.50	94.70	41.50	2.10
W737	M	81	92	28.28	1.54	9.17	19.37	127.92	123.28	27.59	80.10	56.71	49.80	97.10	48.40	2.40
W738	M	100	105.8	41.8	1.72	9.67	25.33	154.27	149.68	24.63	80.55	64.13	63.20	73.10	21.00	2.40
W739	M	100	110.6	44.86	2.36	6.17	24.10	146.10	133.24	23.69	79.56	62.05	61.60	34.20	8.90	2.00
W740	M	81	85.8	29.18	1.52	14.00	26.22	253.15	230.23	29.85	81.05	63.60	58.50	86.00	39.50	2.20
W741	M	85	86.6	34.18	1.54	10.83	19.78	82.93	71.67	30.37	76.67	57.31	52.30	93.40	45.20	2.20
W742	M	104	132.8	36.88	2.44	7.50	27.73	262.47	253.33	22.38	79.80	62.17	61.20	12.30	3.10	2.40
W743	M	100	91.4	34.34	1.98	6.83	21.83	135.57	125.77	29.02	78.89	55.50	50.40	40.20	7.90	2.10
W744	M	95	92.8	50.86	1.74	7.00	24.79	133.40	127.07	31.90	77.38	57.72	52.90	27.60	5.50	2.10
W7	R	114	131.2	36.94	2.16	6.00	26.41	218.94	202.63	28.25	77.60	55.92	51.90	1.20	0.30	2.30

Note: M, R and S in the table refer to the maintainer line and restorer line of rice CMS Lines, and special rice. Phenotypic trait number (1 to 15) in first row correspond from left to the right to The period from seeding to heading (d); Plant heights (cm); Leaf length (cm); Leaf width (cm); Average single plant valid spike number; Spike lengths (cm); Kernel numbers per spike; Grain numbe; 1000-seed weights (g); Brown rice rate (100%); Milled rice rate (100%); Head rice rate (100%); Chalky rice rate; Chalkiness; Length-width ratio

**Table 2:** Basic statistical analysis and diversity of the 15 phenotypic traits

Phenotypic traits	Mean±SD	Median	Mode	Rang	*CV* (%)	*H*_*e*_
The period from seeding to heading(d)	102.95±10.543	105	110	47	10.24	1.91
Plant heights (cm)	112.766±14.994	115.600	121.8	65.6	13.30	2.05
Leaf length (cm)	38.6727±7.51025	37.5600	30.12	53.76	19.42	1.89
Leaf width (cm)	2.0423±0.33795	2.0800	1.54	1.44	16.55	2.08
Average single plant valid spike number	7.74±2.007	7.50	6	11	25.83	2.03
Spike lengths (cm)	25.07±2.593	24.98	24	13	10.34	2.02
Kernel numbers per spike	174.42±52.668	165.61	190	358	30.20	1.92
Grain number	159.68±49.365	153.17	176	332	30.91	1.92
1000-seed weights (g)	26.75±4.129	27.12	20	17	15.44	2.06
Brown rice rate (100%)	78.76±2.414	78.85	79	23	3.07	1.79
Milled rice rate (100%)	58.80±4.439	59.42	67	19	7.59	2.04
Head rice rate (100%)	53.61±8.955	56.20	59	51	16.70	1.89
Chalky rice rate	32.17±31.064	19.90	1	99	96.56	1.68
Chalkiness	11.97±16.45	5	0	80	137.43	1.55
Length-width ratio	2.39±0.293	2.40	2	1	12.26	1.96

Most phenotypic traits were correlated or significantly correlated. The most significant correlation was between kernel numbers per spike and grain number, followed by that between chalky rice rate and chalkiness. However, the correlation between chalky rice rate and length-width ratio was the least significant, followed by that between leaf width and average single plant valid spike number (Table 3)

**Table 3:** Pearson correlation coefficient analysis of the 15 phenotypic traits

Traits	1	2	3	4	5	6	7	8	9	10	11	12	13	14	15
1	1														
2	0.726^**^	1													
3	0.224^*^	0.249^*^	1												
4	0.567^**^	0.582^**^	.261^*^	1											
5	-0.358^**^	-0.398^**^	-0.394^**^	-0.564^**^	1										
6	0.574^**^	0.634^**^	0.398^**^	0.571^**^	-0.469^**^	1									
7	0.268^**^	0.391^**^	0.509^**^	0.460^**^	-0.520^**^	0.554^**^	1								
8	0.260^*^	0.393^**^	0.501^**^	0.428^**^	-0.519^**^	0.545^**^	0.984^**^	1							
9	0.179	0.111	0.074	0.159	-0.024	0.147	-0.272^**^	-0.250^*^	1						
10	-0.107	-0.130	0.098	-0.013	0.156	-0.116	0.085	0.067	0.078	1					
11	0.053	0.095	0.049	0.014	0.222^*^	-0.037	-0.014	-0.022	0.012	0.336^**^	1				
12	0.076	0.125	0.058	0.055	0.115	-0.054	0.031	0.065	-0.045	0.155	0.680^**^	1			
13	-0.491^**^	-0.414^**^	-0.053	-0.322^**^	0.152	-0.268^**^	-0.158	-0.140	0.145	0.077	-0.239^*^	-0.270^**^	1		
14	-0.466^**^	0.353^**^	-0.017	-0.301^**^	0.116	-0.240^*^	-0.116	-0.099	0.036	-0.143	-0.295^**^	-0.208^*^	0.891^**^	1	
15	0.095	0.171	0.005	-0.015	0.156	0.104	-0.047	-0.084	-0.042	0.001	0.360^**^	0.134	-0.604^**^	-0.518^**^	1

*Note*: Asterisk indicates significant difference between phenotypic traits using two-tailed t-tests. *P<0.05; **P<0.01

Principal components were extracted based on the criterion that the eigenvalue was greater than 1.0. The eigenvalues of the first four PCs in 15 phenotypic traits were greater than 1.0, and together accounted for 70.693% of the phenotypic variation (Table 4). The first PC accounted for 31.527%; the most important traits were spike lengths (0.167), plant heights (0.165) and leaf width (0.159). The second PC accounted for 18.137%, the most important traits being length-width ratio (0.252), milled rice rate (0.244) and head rice rate (0.195)

#### Phenotypic traits clustering

**Fig. 1:**
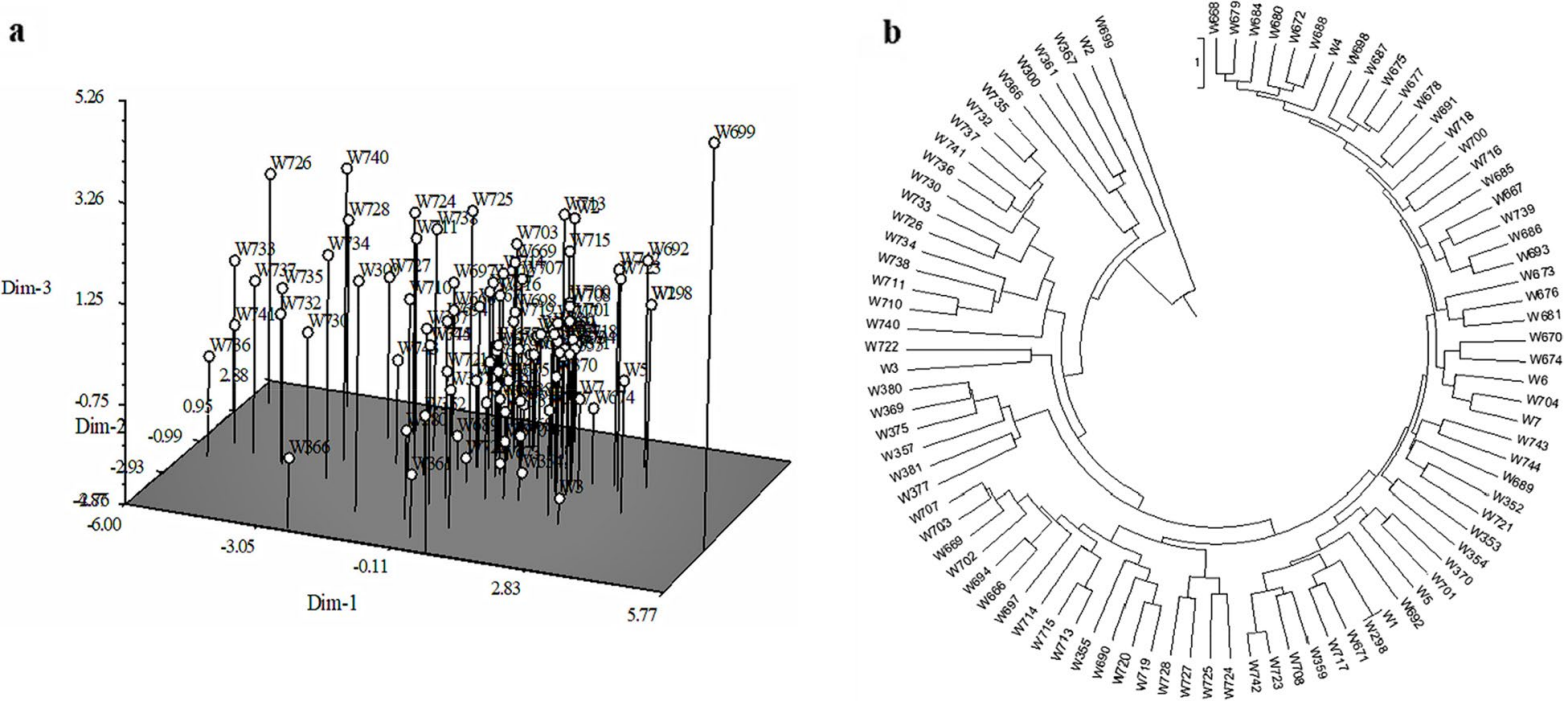
Cluster diagram based on the 15 phenotypic traits. **a** via PC clustering; **b** via UPGMA clustering

Average Euclidean distance was 5.19, ranging from 0.90 (between W723 and W742) to 13.73 (between W699 and W733). Clustering result based on the 15 phenotypic traits was shown in Fig. 1, which demonstrated that the 92 samples were clustered together in addition to W669 and showed a single genetic basis for the population.

## SSR marker analysis

### Polymorphism of SSR markers

A total of 378 bands was detected using 48 core SSRs primer pairs (Table 5). Among these, 336 polymorphic bands were detected. The average number of polymorphic fragments was 7, ranging from 1 to 14. The highest number (14) of polymorphic bands was detected by RM278 while RM311 is the least bands. The average value of PPB (Percentage of polymorphic bands) was 88.87%, ranging from 50% to 100%. The average value of PIC (Polymorphism information content) was 0.77, ranging from 0.19 to 0.88. Data showed that core SSR in rice can produce rich bands and high polymorphic rate.

**Table 4:** Eigenvalue and contributive percentage of principal components and component scores coefficient matrix of the 15 phenotypic traits

Traits code	First principal component	Second principal component	Third principal component	Fourth principal component
The period from seeding to heading(d)	0.155	0.052	-0.212	0.135
Plant heights (cm)	0.165	0.029	-0.138	0.106
Leaf length (cm)	0.108	-0.098	0.188	0.130
Leaf width (cm)	0.159	-0.038	-0.091	0.156
Average single plant valid spike number	-0.136	0.155	0.050	0.020
Spike lengths (cm)	0.167	-0.066	-0.083	0.081
Kernel numbers per spike	0.155	-0.136	0.262	-0.151
Grain number	0.152	-0.141	0.264	-0.137
1000-seed weights (g)	0.001	0.002	-0.224	0.565
Brown rice rate (100%)	-0.007	0.071	0.305	0.267
Milled rice rate (100%)	0.023	0.244	0.279	0.232
Head rice rate (100%)	0.030	0.195	0.272	0.194
Chalky rice rate	-0.121	-0.242	0.099	0.244
Chalkiness	-0.110	-0.248	0.077	0.142
Length-width ratio	0.043	0.252	-0.039	-0.194
Eigenvalue	4.729	2.721	1.763	1.391
Contributive percentage (%)	31.527	18.137	11.756	9.272
Cumulative contributive percentage (%)	31.527	49.665	61.420	70.693

**Table 5:** Information and polymorphism of 48 SSR primers

Primer name	Chr.	Sequence(5’-3’)	Annealing temperature (°C)	TNB	NPB	PPB (%)	PIC
RM583	1	F:agatccatccctgtggagag; R:gcgaactcgcgttgtaatc	55	10	10	100	0.86
RM71	2	F:ctagaggcgaaaacgagatg; R:gggtgggcgaggtaataatg	55	8	8	100	0.84
RM85	3	F:ccaaagatgaaacctggattg; R:gcacaaggtgagcagtcc	55	9	9	100	0.85
RM471	4	F:acgcacaagcagatgatgag; R:gggagaagacgaatgtttgc	55	8	6	75	0.86
RM274	5	F:cctcgcttatgagagcttcg; R:cttctccatcactcccatgg	55	12	12	100	0.84
RM190	6	F:ctttgtctatctcaagacac; R:ttgcagatgttcttcctgatg	55	5	5	100	0.74
RM336	7	F:cttacagagaaacggcatcg; R:gctggtttgtttcaggttcg	55	7	7	100	0.79
RM72	8	F:ccggcgataaaacaatgag; R:gcatcggtcctaactaaggg	55	12	9	75	0.86
RM219	9	F:cgtcggatgatgtaaagcct; R:catatcggcattcgcctg	55	2	2	100	0.36
RM311	10	F:tggtagtataggtactaaacat; R:tcctatacacatacaaacatac	55	2	1	50	0.37
RM209	11	F:atatgagttgctgtcgtgcg; R:caacttgcatcctcccctcc	55	4	3	75	0.67
RM19	12	F:caaaaacagagcagatgac; R:ctcaagatggacgccaaga	55	12	9	75	0.86
RM1195	1	F:atggaccacaaacgaccttc; R:cgactcccttgttcttctgg	55	8	8	100	0.84
RM208	2	F:tctgcaagccttgtctgatg; R:taagtcgatcattgtgtggacc	55	5	4	80	0.75
RM232	3	F:ccggtatccttcgatattgc; R:ccgacttttcctcctgacg	55	10	10	100	0.87
RM119	4	F:catccccctgctgctgctgctg; R:cgccggatgtgtgggactagcg	67	7	4	57.14	0.79
RM267	5	F:tgcagacatagagaaggaagtg; R:agcaacagcacaacttgatg	55	9	5	56.56	0.85
RM253	6	F:tccttcaagagtgcaaaacc; R:gcattgtcatgtcgaagcc	55	6	6	100	0.75
RM481	7	F:tagctagccgattgaatggc; R:ctccacctcctatgttgttg	55	7	7	100	0.80
RM339	8	F:gtaatcgatgctgtgggaag; R:gagtcatgtgatagccgatatg	55	8	8	100	0.79
RM278	9	F:gtagtgagcctaacaataatc; R:tcaactcagcatctctgtcc	55	14	14	100	0.85
RM258	10	F:tgctgtatgtagctcgcacc; R:tggcctttaaagctgtcgc	55	7	6	85.71	0.80
RM224	11	F:atcgatcgatcttcacgagg; R:tgctataaaaggcattcggg	55	8	8	100	0.84
RM17	12	F:tgccctgttattttcttctctc; R:ggtgatcctttcccatttca	55	9	9	100	0.78
RM493	1	F:tagctccaacaggatcgacc; R:gtacgtaaacgcggaaggtg	55	7	7	100	0.83
RM561	2	F:gagctgttttggactacggc; R:gagtagctttctcccacccc	55	8	5	62.50	0.85
RM8277	3	F:agcacaagtaggtgcatttc; R:atttgcctgtgatgtaatagc	55	7	7	100	0.75
RM551	4	F:agcccagactagcatgattg; R:gaaggcgagaaggatcacag	55	6	6	100	0.68
RM598	5	F:gaatcgcacacgtgatgaac; R:atgcgactgatcggtactcc	55	9	5	55.56	0.75
RM176	6	F:cggctcccgctacgacgtctcc; R:agcgatgcgctggaagaggtgc	67	10	7	70	0.88
RM432	7	F:ttctgtctcacgctggattg; R:agctgcgtacgtgatgaatg	55	5	5	100	0.71
RM331	8	F:gaaccagaggacaaaaatgc; R:catcatacatttgcagccag	55	8	7	87.50	0.82
OSR28	9	F:agcagctatagcttagctgg; R:actgcacatgagcagagaca	55	10	9	90	0.80
RM590	10	F:catctccgctctccatgc; R:ggagttggggtcttgttcg	55	9	6	66.67	0.87
RM21	11	F:acagtattccgtaggcacgg; R:gctccatgagggtggtagag	55	11	11	100	0.87
RM3331	12	F:cctcctccatgagctaatgc; R:aggaggagcggatttctctc	50	6	4	66.67	0.80
RM443	1	F:gatggttttcatcggctacg; R:agtcccagaatgtcgtttcg	55	10	7	70	0.75
RM490	1	F:atctgcacactgcaaacacc; R:agcaagcagtgctttcagag	55	9	9	100	0.82
RM424	2	F:tttgtggctcaccagttgag; R:tggcgcattcatgtcatc	55	5	5	100	0.72
RM423	2	F:agcacccatgccttatgttg; R:cctttttcagtagccctccc	55	7	7	100	0.82
RM571	3	F:ggaggtgaaagcgaatcatg; R:cctgctgctctttcatcagc	55	7	7	100	0.67
RM231	3	F:ccagattatttcctgaggtc; R:cacttgcatagttctgcattg	55	12	12	100	0.84
RM567	4	F:atcagggaaatcctgaaggg; R:ggaaggagcaatcaccactg	55	10	10	100	0.78
RM289	5	F:ttccatggcacacaagcc; R:ctgtgcacgaacttccaaag	55	10	10	100	0.88
RM542	7	F:tgaatcaagcccctcactac; R:ctgcaacgagtaaggcagag	55	8	7	87.50	0.84
RM316	9	F:ctagttgggcatacgatggc; R:acgcttatatgttacgtcaac	55	2	2	100	0.19
RM332	11	F:gcgaaggcgaaggtgaag; R:catgagtgatctcactcaccc	55	10	8	80	0.88
RM7102	12	F:taggagtgtttagagtgcca; R:tcggtttgcttatacatcag	55	3	3	100	0.43

## Clustering based on SSR

**Fig. 2:**
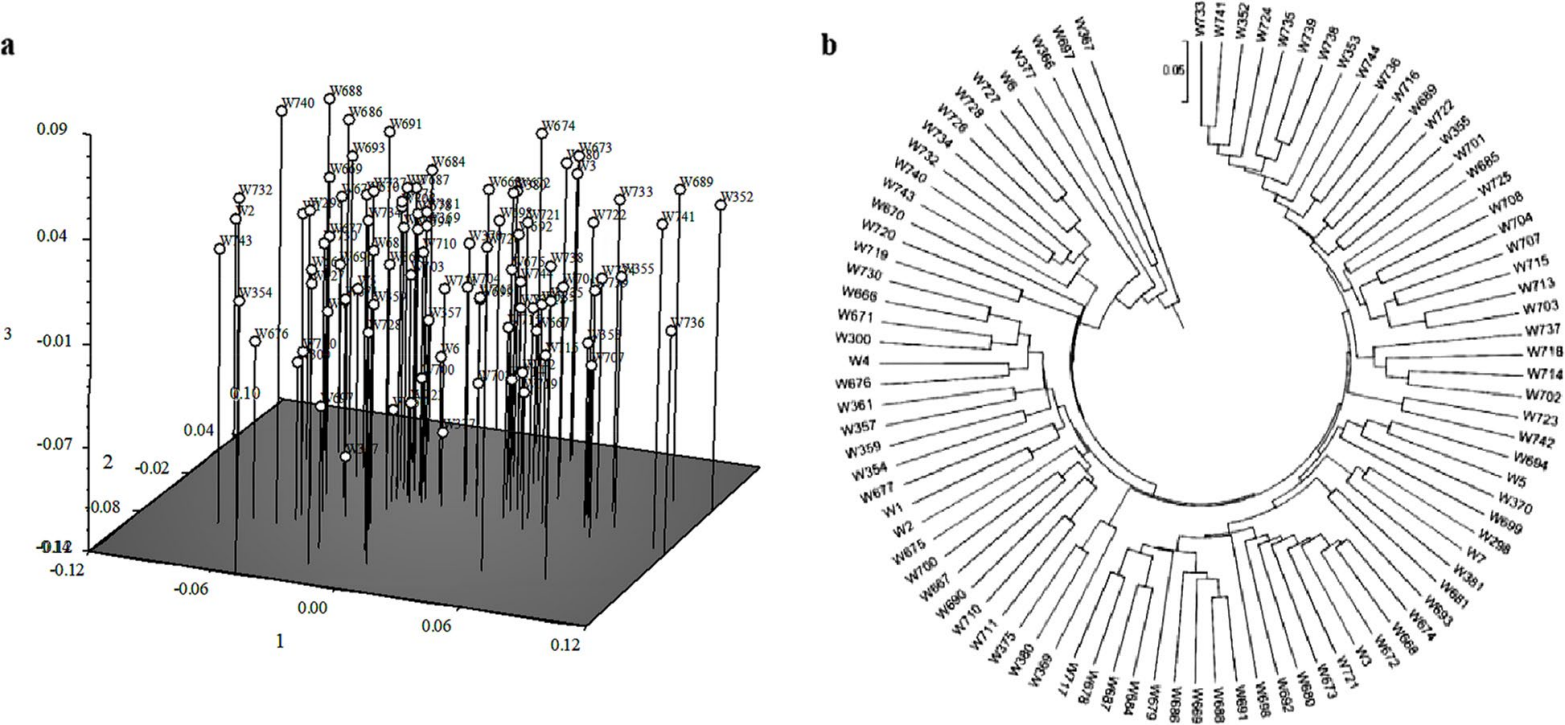
The cluster diagram based on SSR. **a** by the PC clustering; b by the UPGMA clustering

PC, in which the first three PC (eigenvalue) to select and their cumulative contribution of variance accounted for 15.76%, and the unweighted pair-group method with arithmetic means (UPGMA) were performed, which demonstrated that the 93 genotypes could be divided into 2 subgroups (Fig. 2).

## Bayesian clustering based on SSR markers

**Fig. 3:**
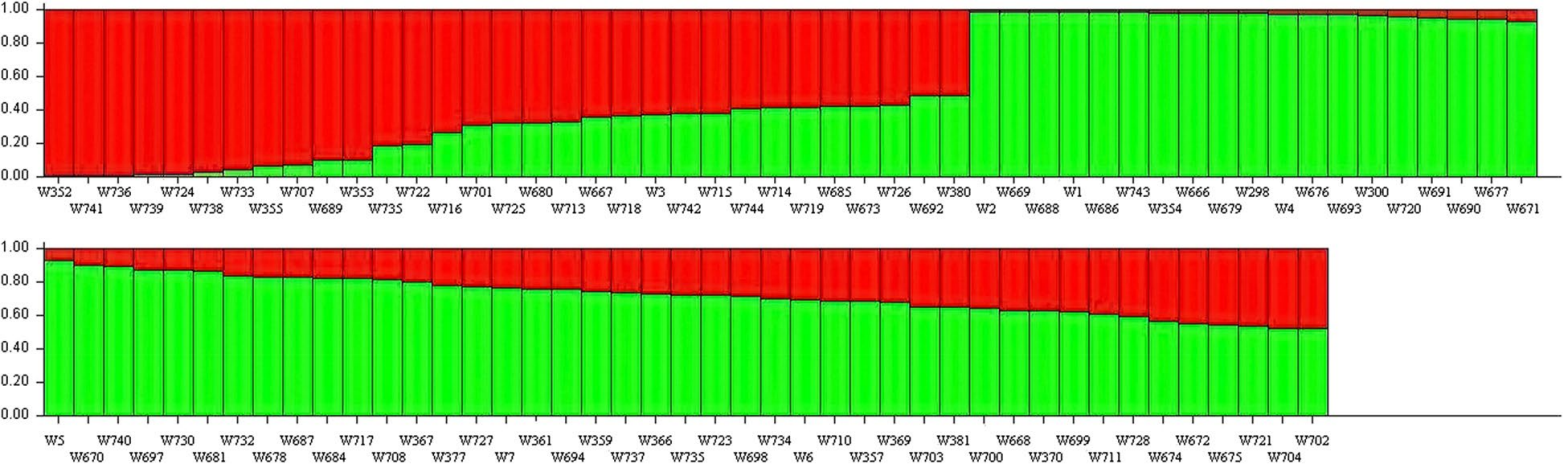
Bayesian clustering based on SSR markers; Red: group I; Green: group II. Each vertical line on the X-axis correspond to a sample. The proportion of each color represents probability rate with which a given genotype belongs to each group

A total of 378 SSR bands was used to elucidate the population structure of the entire pool of 93 rice germplasms. The best K was K = 2, suggesting that the 93 rice germplasms were best divided into two subgroups (Fig. 3).

## SNPs marker analysis

A total of 39,872 SNPs_*-Nla*III_ and 35,547 SNPs_*-Mse*I_ passed the minor allele frequency (MAF) lower limit of 0.05 using *Nla*III and *Mse*I digestion, respectively. Merged data of SNPs_*-Nla*III_ and SNPs_*-Mse*I_, with a total of 72,824 SNPs including 67,621 SNPs that aligned to specific chromosomes and 5,023 SNPs unlocalized, were then obtained.

## Linkage disequilibrium (LD) and haplotype analysis

**Fig. 4:**
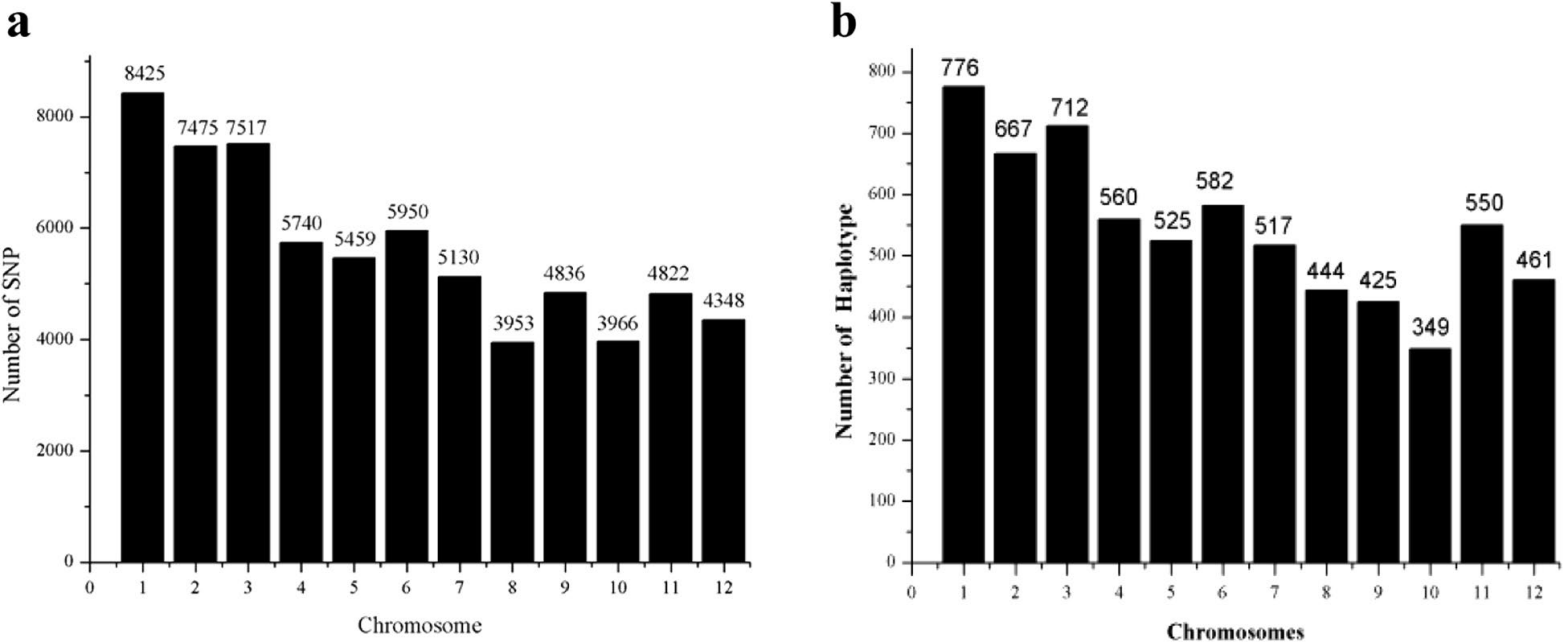
The distribution of SNPs and haplotype among the 12 chromosomes. **a** SNPs distribution. **b** Haplotype distribution

From the total of 6,288,753 loci (93 samples × 67,621SNPs), 326,873 (5.198%) were heterozygous. The 67,621 SNPs were unevenly distributed on the 12 chromosomes (Fig. 4a); chromosome 1 contained the largest number of makers (8,425), while chromosome 8 included the least (3,953). LD, as represented by inter-loci *R*^*2*^ values, was calculated for the 84,255 SNP pairs. *R*^*2*^ value had a minimum of 0.2 and an average of 0.73. 46,322 SNP pairs (54.98%) had *R*^*2*^ values higher than 0.8, while 7,841 pairs (9.31%) were in complete LD (*R*^*2*^=1). The 12 chromosomes yielded a total of 6,568 predicted haplotypes (Fig. 4b), with chromosome 1 possessing the most haplotypes (776) and chromosome 10 possessing the least (349). The largest haplotype was composed of 95 SNPs. The longest haplotype spanned over 200.0 kb; the average haplotype length was 33.71kb.

## AMOVA and gene flow

The average MAF of the 93 samples was 0.21. Tajima’s D value was 1.66, which suggests low levels of both low- and high-frequency polymorphisms, indicating a decrease in population size and/or balancing selection that resulted in more haplotypes and lacked rare alleles in this population. Analysis of molecular variance (AMOVA) showed that the genetic variation was 98% within the population and 2% between populations, which indicated the existence of slight genetic variation among 93 samples. The genetic differentiation coefficient (*F*_ST_) between the two populations was 0.61, and gene flow (*N*_m_) was 0.16. Further investigation showed that the gene flow of selfing crops was the smallest, and that of annual herbaceous plants was the lowest. If *N*_m_ > 1, which indicates that the level of gene flow between populations is high, then genetic differentiation among populations is small; if *N*_m_ > 4, then gene communication between populations is more adequate and genetic differentiation is smaller; and *N*_m_ < 1 indicates that population differentiation may have occurred due to genetic drift. The gene flow was 0.16, which indicates that the gene flow among populations in the Qinba region is lower, but nearly 2.5-fold higher than that of conventional inbred plants, which may result in long-term artificial selection, leading to reduced genetic differentiation.

## Clustering based on SNPs

### PC clustering

**Fig. 5:**
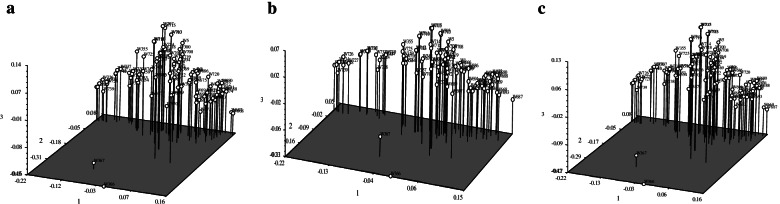
PCA plots using the different SNPs markers. **a** by SNPs_*-Nla*III_; **b** by SNPs_*-Mse*I_; **c** by merged SNPs_*-Nla*III_ and SNPs_*-Mse*I_ data

Principal component analysis was performed to select the first three PCs (based on eigenvalue). Their cumulative contribution of variance accounted for 40.69%, 39.76% and 40.10% for SNPs_*-Nla*III_, SNPs_*-Mse*I_, merged data of SNPs_*-Nla*III_ and SNPs_*-Mse*I_, respectively, which demonstrated that the 93 genotypes could be clustered into two subgroups by the first three PCs (Fig. 5), with W366 and W367 being always separated from other samples.

## UPGMA clustering

**Fig. 6:**
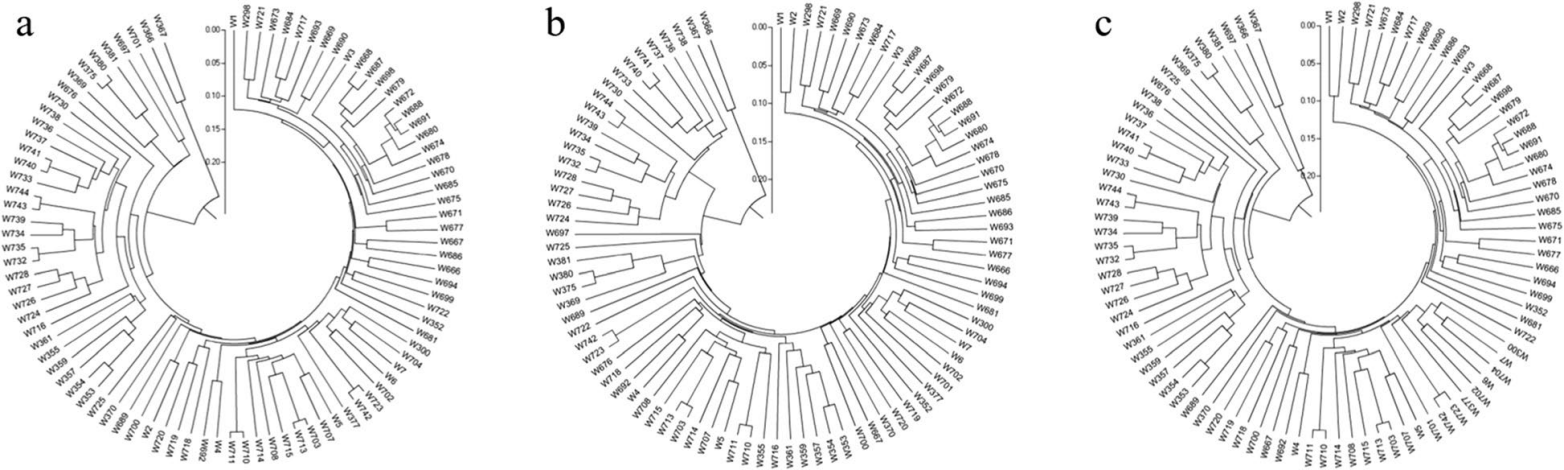
UPGMA Clustering using different SNPs markers. **a** by SNPs_*-Nla*III_; **b** by SNPs_*-Mse*I_; **c** by merged datas of SNPs_*-Nla*III_ plus SNPs_*-Mse*I_

The unweighted pair-group method with arithmetic means (UPGMA) algorithm was performed and demonstrated that the 93 genotypes could be divided into 2 subgroups (Fig. 6), which was consistent with the PC results. Group I included 1 to 3 samples, while group II contained 92 to 90 samples. The average genetic distance was 0.29, ranging from 0.02 to 0.55 based on merged SNPs_*-Nla*III_ and SNPs_*-Mse*I_ data. The two most closely related materials were W710 and W711, and the two most furthest materials were W366 and W740.

## Bayesian clustering

**Fig. 7:**
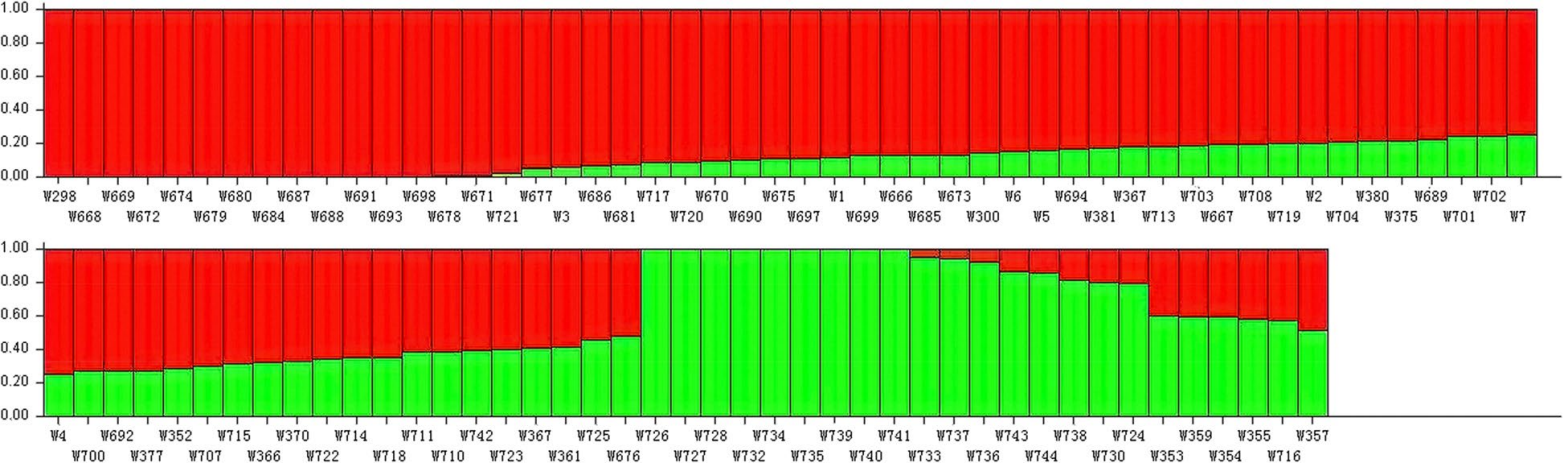
Bayesian clustering based on 72,824 SNPs for 93 samples; Red: group I; Green: group II. Each vertical line on the X-axis correspond to a sample. The proportion of each color represents probability rate with which a given genotype belongs to each group

Seventy-two thousand eight hundred twenty-four SNPs (MAF <5%) were used to assess the population structure of the entire pool of 93 samples. Delta K reached a maximum value at K=2, suggesting that the 93 samples were divided into two subgroups (consisting of 70 and 23 samples) (Fig. 7). In the population structure analysis, the results from K = 2 to K = 5 revealed the occurrence of gene introgression between groupІ and groupII, accounting for approximately 76.34% of the observed variations (calculated with K = 2).

The analysis performed based on phenotypic traits, SSR and SNPs data using PCA, UPGMA and Bayesian clustering led to inconsistent results, the population structure is relatively simple, the matrix delamination is not distinctive.

## Clustering of different category materials

**Fig. 8:**
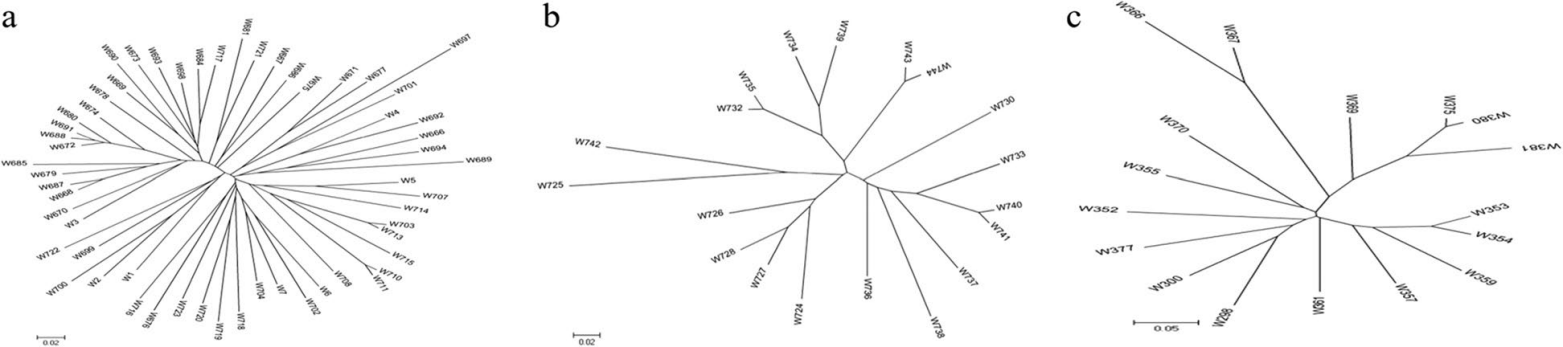
Clustering based on UPGMA. **a** clustering of 57 restoring lines. **b** clustering of 19 maintainer lines. **c** clustering of 17 special rice lines

Population genetic information of different category samples, including 57 restoring lines, 19 maintainer lines and 17 special rice lines was analyzed (Table 6) and clustered (Fig 8a, b, c, respectively). Results showed that the genetic basis of the restorer line was more abundant than that of the maintainer line, and that the genetic basis of the special rice was wider than that of the conventional rice.

**Table 6:** Population genetic analysis of different category materials

Samples	Tajima’ D	Range of IBS genetic distance	The average genetic distance	Two samples with the closest genetic distance	Two samples with the farthest genetic distance
Whole materials (93)	1.66	0.0229-0.5452	0.3007	W710/W711	W366/W740
Restoring lines (57)	1.36672	0.0229-0.3927	0.2666	W710/W711	W685/W697
Maintainer lines (19)	0.43533	0.0242-0.3745	0.2293	W740/W741	W725/W738
Special rice (17)	0.62542	0.0285-0.5315	0.3280	W375/W380	W300/W366

## Correlation analysis of genetic distance matrices based on 3 types of genetic markers

**Fig. 9:**
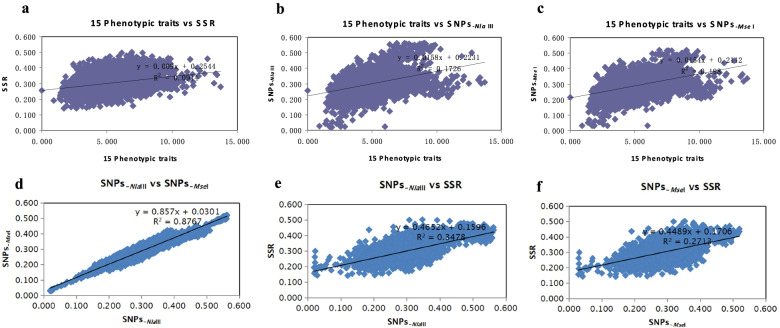
Correlation between the genetic distance matrices generated using different genetic markers

All cluster analyses were based on the genetic distance or genetic similarity coefficient generated by genetic markers between samples; in the present study, the coefficients of correlation (*R*^*2*^) between the genetic distance matrices were 0.0914, 0.1726, 0.198, 0.876, 0.3478, 0.2713, respectively (Fig. 9). These results may be due to the use of different number of markers.

## Discussion

Phenotype is the result of the interaction of genotype with environment. A given genotype can be expressed as different phenotypes in different environments; this is known as the plant inherent phenotypic plasticity, which is different from the genotype. In the study of germplasm resources with more samples, economical and effective method with the use of phenotype data to study population genetic structure and genetic diversity is also a very important at this early stage. The selected 15 traits belong to quantitative traits and are greatly affected by environment; hence it is not recommended to use them for population genetic structure analysis. In recent decades, SSR markers, which represent second-generation DNA molecular markers, have been widely used for plant population genetic analysis, phylogenetic reconstruction, and quantitative trait mapping. All kinds of DNA markers are different and results generated by different DNA markers reflect different polymorphic region in the genome and can reveal various information contained in the genome. Theoretically, the more markers used, the more accurate results will be. SSR markers are mostly distributed in centromeres, telomeres, introns, and 3' untranslated regions (UTR). Most of these markers are non-functional genetic markers and do not affect the application of SSR marker in clustering analysis. While the population genetic variation explained by SNPs was larger than that explained by SSRs (for example, in PC analysis), the accumulative contribution rate of the first three major factors analyzed by SSR was only 15.76%, far less than that using SNPs data (40.10%), indicating that the more DNA polymorphism, the more accurate the population variation can be explained. For association analysis between markers and traits, greater number of polymorphic sites is associated with higher mapping resolution. A natural population often contains multiple sub-populations, which could result in high degree of LD within the tested population and lead to pseudo-association between markers and traits. Therefore, LD and haplotype studies are necessary before carrying out association analysis. The core 48 pairs of SSR markers as well 72,824 SNPs had rich bands and high polymorphism in Indica rice genome; clustering result of SSR was concordant with that of SNPs, but different from phenotypic traits clustering.

All analyses of the population genetic structure were based on the estimation of genetic distance or genetic similarity coefficient matrix between samples. Analyses were conducted using three methods: PC, UPGMA, and Bayesian clustering. Bayesian algorithm is more practical than UPGMA and PC analysis no matter which genetic marker is used given the prior pedigree knowledge of the 93 samples. At the same time, the size of gene flow of each sample can be seen from the population genetic structure graph based on Bayesian algorithm.

Through the analysis of different types of materials (57 restorer lines, 19 sterile lines, and 17 special rice), the results showed that the genetic basis of the restorer lines was richer than that of the maintainer lines, which was consistent with the conclusion of Ying Jiezheng et al. [24]. The main reason may be that most CMS (cytoplasmic male sterile) lines currently used in production are related to cultivars such as Zhenshan 97B, II-32B, Zhong9a and Gang46a and may be derived from Aizazhan and Aijiaonante, which originated from dwarf rice varieties. At present, the restorer lines used in combination production originate from the Yangtze River Basin of China, Sichuan, Southeast Asia, South Korea, etc, and were created by crossing Indica and Japonica rice. Special rice has an abundant genetic basis compared to other rice germplasm resources and has high breeding potential.

## Conclusions

Higher number of genetic markers is related to higher explained population variation, especially functional DNA markers. The above showed that it is difficult to make certain the genetic nature of rice germ-resources using phenotype traits clustering. Clustering results based on different genetic markers showed that the genetic basis of 93 samples was single. Average genetic distance was 0.29 based on 72,824 SNPs of 93 samples, which may be due to many reasons, such as the wide exchange of variety resources among breeding units in the process of breeding, and similar breeding goals. Genetic effects in populations depend on the opportunity distribution of MAFs across the genome-wide, and different populations have different MAF values. Although the gene flow in the population composed of 93 samples was relatively large, the average MAF of the population was only 0.21, indicating the genetic structure of 93 samples is simple and lacked rare alleles. Though the amount of colored rice only take up a small proportion of rice resource in this study, it arose an extensive attention all over the world, due to its characteristics which include special nutrition, health care and artificial utilization. Measures to improve the genetic diversity of rice cultivars in the Qinba area are important in the future.

## Materials and methods

### Plant materials

A total of 93 samples were collected from the Shaanxi Rice Research Institute (Hanzhong city, China), comprising 57 restoring lines, 19 maintainer lines, and 17 special rice (Special rice refers to rice with special genetic traits and uses such as colored rice genotypes including black rice, purple rice, red rice, green rice and yellow rice and aromatic rice germplasm, which only research colored rice in this study.), which were representative of the diversity of *Oryza sativa* Xian group present in the Qinba area in China.

### Field experiments

Seeds were planted at the rice experimental farm (E: 106°59′57″, N: 33°7′48″) during three consecutive years (2018, 2019, 2020), with planting dates of 2018 April 10, 2019 April 11, and 2020 April 8, and transplanted on May 24, May 24, May 20 according to a 16.7cm × 20cm split-split-plot design. Each sample was arrayed randomly at plots with three repeats, to no edge row between the plots.

### Phenotyping

Six plants in the middle of each plot were selected to investigate the values of agronomic, economic and quality traits according to “Recording items, methods and standards of national rice variety test and observation” as well as “National Standard of GBT 17891-1999 high quality paddy”. The 15 selected phenotypic traits included sowing date, plant height, leaf length, leaf width, effective number of panicles per plant, panicle length, total number of grains per panicle, number of filled grains per panicle, 1000-grain weight, browning rate, milled rice rate, head milled rice rate, chalky grain rate, degree of chalkiness, and length/width ratio; the averages of the three-year data were used as the phenotypic data.

### Phenotypic traits statistical analysis

The mean value (x), standard deviation (δ), and coefficient of variation (*CV*) were computed. Shannon-Weiner index (*H’*) was calculated according to the following equation: *H’=-*∑P_i_lnP_i_, where P_i_ is the proportion of samples ranked at i^th^ grade for a given phenotypic trait among all samples (all of the phenotypic traits were divided into 10 grades by assigning values less than $\overline{\mathrm{x}}\hbox{-} 2\delta$ as 1^th^ grade and those greater than $\overline{\mathrm{x}}+2\delta$ as 10^th^ grade, with inter-grade difference of 0.5δ for the remaining grades). All of phenotypic trait data were the standardized using Z-scores, and hierarchical cluster analysis was performed using between-groups linkage method based on Euclidean distance. The above analysis was carried out with the IBM SPSS statistics 22.0 software; MEGA7.0 software was used for editing and visualizing cluster results.

### SSR genotyping

The genomic DNA of 93 samples was extracted from fresh leaves using the SDS technique and detected with 0.8% agarose gel electrophoresis. The 48 SSR primers were synthesized by Beijing Aoke Biotechnology Co., Ltd. (Beijing, China). PCR were carried out in a 10 μL volume containing 1 μL DNA template, 2 μL (10 μM) of forward and reverse primers (1μL each), 5μL 2×Taq Master Mix, and 2 μL RNase-free water. Reactions were programed as follows: initial denaturation at 94.0°C for 5 minutes, denaturation at 94.0°C for 1 minute, annealing at 50-60.0°C for 1 minute, and extension at 72.0°C for 1 minute, for a total of 35 cycles. Electrophoresis was performed using 8% non-denaturing polyacrylamide gel under 95V voltage; bands were visualized via silver staining. Following electrophoresis, each amplification band corresponded to a primer hybridization locus and was considered as an effective molecular marker. Each polymorphic band detected by a same given primer represented an allelic mutation. In order to generate molecular data matrices, clear bands for each fragment were scored in every accession for each primer pair and recorded as 1(presence of a fragment), 0(absence of a fragment), and 9(complete absence of band).

### SSR marker efficiency analysis

The value of the polymorphism information content (PIC) was calculated using the PIC_Calc 0.6 program (http://www.bio-soft.net/dna/pic.htm). The level of polymorphism of each marker was assessed by the polymorphism information content, which measures the extent of genetic variation: PIC values smaller than 0.25 indicates low levels of polymorphism associated to a locus, PIC values between 0.25 and 0.5 imply moderate levels of polymorphism, while PIC values greater than 0.5 indicate high levels of polymorphism [25].

### SNPs genotyping

The genomic DNA of 93 samples was digested using the *Nla*III and *Mse*I enzymes. GBS was performed using the Illumina Hiseq 2000 platform of Novo Gene Bioinformatics Technology Co.,Ltd (Beijing, China). The SNPs data obtained with *Nla*III and *Mse*I digestion were recorded as SNPs_-*Nla*III_, SNPs_-*Mse*I_, respectively. Polymorphism filtering of SNPs was done with dp., Miss and MAF of 2, 0.3 and 0.05, respectively, followed by annotation based on the reference genome (ftp://ftp.ensemblgenomes.org/pub/plants/release-37/fasta/oryza_indica/dna/).

### LD and haplotype construction

Genotype data were then used to calculate LD between SNPs and to construct haplotypes using the EM algorithm implemented in PLINK1.07 (https://www.cog-genomics.org/plink2). The commands “--r2” and “--blocks” were used to calculate LD and assign SNPs to their respective haplotypes by calculating inter-maker LD within a 200kb window, respectively. Figures were constructed using the Origin8 platform (http://www.originlab.com/).

### AMOVA and gene flow

A total of 72,824 SNPs were employed to analyze molecular variance (AMOVA) and gene flow. The components of variance attributable to different varieties and breeding lines were estimated from the genetic distance matrix using the Tajima & Nei method, as specified in the AMOVA procedure in ARLEQUIN 3.1 [26]. A nonparametric permutation procedure with 9999 permutations was used to test the significance of variance components associated with the different possible levels of genetic structure in this study. The pairwise *Fst* values, a value of F statistic analogs computed from AMOVA, were used to compare genetic distances between any two groups.

### PC clustering

PC analysis was performed under the Eigen module using NTSYS-pc2.10e [27].

### UPGMA clustering

Identity-by-state (IBS) distance matrix generated by TASSEL5.0 (http://www.maizegenetics.net/tassel) was used to build an UPGMA tree. MEGA7.0 (http://http://www.megasoftware.net/) was used for editing and visualizing.

### Bayesian clustering

STRUCTURE 2.3.4 (http://taylor0.biology.ucla.edu/structureHarvesteroybase.org/tools.php), which applies a Bayesian clustering algorithm, was used to simulate population genetic structure based on SSR and SNPs data, respectively. Using a membership probability threshold of 0.60, population K values from 1 to 5 were simulated with 5 iterations for each K using 10,000 burn-in periods followed by 10,0000 Markov Chain Monte Carlo iterations in order to obtain an estimate of the most probable number of populations. Delta K was plotted against K values; the best number of clusters was determined following the method proposed by Evanno et al [28], and obtained via the Structure Harvester platform (http://taylor0.biology.ucla.edu/structureHarvester/) [29].

### Correlation analysis among genetic distance matrices by diffrent DNA marker dataset

Mantel tests were used to measure the correlation between the genetic distance matrices generated using 15 phenotypic traits and SSR, 15 phenotypic traits and SNPs_*-Nla*III_, 15 phenotypic traits and SNPs_*-Mse*I_, SNPs_*-Nla*III_ and SNPs_*-Mse*I,_ SNPs_*-Nla*III_ and SSRs, SNPs_*-Mse*I_ and SSRs. It was carried out using the GenAlEx software with 9999 permutations [30]. r ≥ 0.9, 0.8 ≤ r < 0.9, 0.7 ≤ r < 0.8, and r < 0.7 represented significant correlation, moderate correlation, weak correlation, and no correlation, respectively.

## Data Availability

The datasets generated during the current study are available in the NCBI repository, [https://www.ncbi.nlm.nih.gov/bioproject/PRJNA801889] [PRJNA801889].

## Abbreviations

AMOVA: Analysis of molecular variance
DNA: Deoxyribonucleic acid
GWAS: genome-wide association studies
GBS: genotyping by sequencing
IBS: Identity by state
LD: Linkage disequilibrium
MAF: Minor allele frequency
NPB: number of polymorphic bands
PPB: Percentage of polymorphic bands
PIC: Polymorphism information content
PCA: Principal component analyses
RAD: restriction site-associated DNA
SNP: Single nucleotide polymorphism
SSR: Simple sequence repeats
TNB: total number of bands
UPGMA: Unweighted pair group method with arithmetic mean
